# An Impact Assessment of Beach Wrack and Litter on Beach Ecosystem Services to Support Coastal Management at the Baltic Sea

**DOI:** 10.1007/s00267-021-01533-3

**Published:** 2021-09-09

**Authors:** Esther Robbe, Jana Woelfel, Arūnas Balčiūnas, Gerald Schernewski

**Affiliations:** 1 Coastal and Marine Management Group, Leibniz Institute for Baltic Sea Research Warnemünde, Rostock, Germany; 2 Marine Research Institute, Klaipeda University, Klaipeda, Lithuania; 3 Institute of Biological Sciences, Aquatic Ecology, University of Rostock, Rostock, Germany

**Keywords:** Beach cleaning, Marine litter, Beach cast, Expert-based, Stakeholder participation, Online assessment

## Abstract

As accumulation zones, sandy beaches are temporal sinks for beach wrack and litter, both often seen as nuisances to tourists. Consequently, there is a need for beach management and an enhanced political interest to evaluate their ecosystem services. We applied a new online multidisciplinary assessment approach differentiating between the provision, potential, and flow at German and Lithuanian beaches (Southern Baltic Sea). We selected a set of services and assessed four beach scenarios developed accordingly to common management measures (different beach wrack and litter accumulations). We conducted comparative assessments involving 39 external experts using spread-sheets and workshops, an online survey as well as a combined data-based approach. Results indicated the relative importance of cultural (52.2%), regulating and maintenance (37.4%), and provisioning services (10.4%). Assessed impact scores showed that the removal of beach wrack is not favorable with regard to the overall ecosystem service provision. Contrarily, the removal of litter can increase the service flow significantly. When removing beach wrack, synergies between services should be used, i.e., use of biomass as material or further processing. However, trade-offs prevail between cultural services and the overall provision of beach ecosystem services (i.e., coastal protection and biodiversity). We recommend developing new and innovative beach cleaning techniques and procedures, i.e., different spatio-temporal patterns, e.g., mechanical vs. manually, daily vs. on-demand, whole beach width vs. patches. Our fast and easy-to-apply assessment approach can support decision-making processes within sustainable coastal management allowing us to show and compare the impacts of measures from a holistic ecosystem services perspective.

## Introduction

Increasing human activities on beaches and developments in the surrounding area have led to the endangerment and often destruction of the typical flora and fauna in recent decades and even centuries (Davenport and Davenport 2006). At first glance, sandy beaches seem almost devoid of faunal life, as animals are often too small to be seen by the naked eye (Radziejewska et al. 2017). Thus often neglected, sandy beaches are important habitats that support a variety of life ranging from microbes to invertebrates and shorebirds (Dahl 1952; McLachlan 1983; Little 2000). Similarly, given the low species diversity of sandy beach vegetation, they harbor a disproportionate amount of rare and endangered species that are adapted to stressful habitat conditions (García‐Mora et al. 1999; Acosta et al. 2009). However, Baltic coasts, especially sandy shores, are mainly related to tourism and recreation and face several human pressures. While beach tourism increased Baltic-wide by 10.4% or 88 million tourist arrivals between 2014 and 2016 (BSTC 2018), large sections of the Baltic coasts account for an annual coastal erosion of 0.2–0.3 m/year on average with the highest loss rates up to 1.5 m/year (Jensen and Schwartzer 2013). Increasing policy relevance and demand for nature protection areas (e.g., Natura 2000), as well as a tourism-driven requirement for beach cleaning, lead to trade-offs between nature conservation and tourism interests. Although many far-reaching impacts of human activity on the beach ecosystem are assumed, sufficient ecological studies which explicitly address this complex topic for the Baltic Sea coast are lacking (Mossbauer et al. 2012; Chubarenko et al. 2021). Spatial conflicts and trade-offs call for consensus-building and decision-making, and thus for coastal management that more holistically integrates human and environmental interests.

A major issue for beach management performed seasonally by the municipalities/resorts at sandy beaches is the accumulation of beach wrack and litter, as they represent nuisances to beach goers (Corraini et al. 2018). As there is no common international definition nor terminology of beach wrack, we defined it as seaborne organic material including seaweed debris (seagrass, macroalgae), remains of dead animals like crabs, and seashells washed ashore. Other terms used in literature include “beach cast”, “beach debris”, and “flotsam” (Liu et al. 2019), or further divided into “terrestrial debris” (Chubarenko et al. 2021). In the Baltic, beach wrack mainly consists of seagrass and macroalgae with only a little amount of shells (Chubarenko et al. 2021). The Køge Municipality in Denmark removed 14,000 t beach wrack year^−1^, while on average it sums up to 1,400–2,800 t year^−1^ (Chubarenko et al. 2021). In Southern Sweden, they determined 57,000–61,000 t, in Solrød municipality (Køge Bight, Denmark) 13,000–24,000 t, and in Sopot Municipality (Gulf of Gdansk, Poland) 160–800 t year^−1^ (Schultz-Zehden and Matczak 2012). Composition and amounts of beach wrack differ highly among coasts due to different hydrodynamics (e.g., currents, wind-driven transport) and offshore vegetation (e.g., seagrass meadows) as well as among season, years, and countries. Despite lacking data on concrete numbers, it still identifies beach wrack as a major problem for local municipalities and their beach management.

In addition, marine litter further intensifies the management problem and complicates the handling of collected material during cleanings by its entanglement within beach wrack. Marine litter is defined as “any persistent, manufactured or processed solid material which has been deliberately discarded, or unintentionally lost onshore or at sea” including plastics as evidently the most dominant group (OSPAR Commission 2010). Others also include feces and organic material, like food waste. Here, we defined litter as a material with anthropogenic origin washed ashore from the sea as well as litter from human activities from the beach, sea-based and land-based sources; we considered only meso (5–25 mm) and macro litter (>25 mm) (Hartmann et al. 2019). Litter pollution is a common problem at sandy beaches, ranging from 0.09 items m^−2^ to 0.61 items m^−1^ and 0.91 items m^−2^ in the Mediterranean mainly composing of plastics (Silc et al. 2018; Asensio-Montesinos et al. 2020; Prevenios et al. 2018) while showing a mean value of 47 to 222 items 100 m^−1^ in the Baltic (Schernewski et al. 2017). For decades, marine litter has been a prevailing and ubiquitous topic within political agendas. Several initiatives and programs included marine litter, for example, the United Nations Environmental Program to achieve their “Sustainable Development Goals”. The European Union defined marine litter as one out of 11 descriptors of the aimed “Good Environmental Status” by the Marine Strategy Framework Directive. For the Baltic Sea, the Helsinki Commission (HELCOM) included marine litter in its “Baltic Sea Action Plan”. Despite its relevance, local municipalities are still missing clear regulations and recommendations for tackling mixed beach wrack with litter, demanding clear thresholds, reduction, and mitigation measures to fight marine litter pollution.

Scarce studies indicated high costs for beach cleanings as an important problem for local municipalities in the Baltic Sea Region (Hofmann and Banovec 2021; Mossbauer et al. 2012; Weinberger et al. 2020). For example, according to Chubarenko et al. (2021), the small municipality of the Island of Poel (Germany) with ca. 2,500 inhabitants treated an average of 3,000 m^3^ of beach wrack per year, resulting in annual costs of 200,000 €. The bigger municipality of Greve (Denmark) with ca. 50,000 inhabitants has paid 268,000 € in 2017 for beach clean-up. However, studies on the beach management costs in the Baltic Sea region, especially on the international level, are rare. Besides, numbers are also hardly comparable, as municipalities face different cleaning conditions in terms of cleaning technique, labor intensity, personnel costs, infrastructure, machinery, tourism density, and amounts of wrack. According to a recent beach wrack study by Hofmann and Banovec (2021), municipalities and private beach operators invest between 20€ and 40€ per m of beach length annually in beach cleaning efforts. However, there is also the loss of income in tourism caused by beach wrack and litter presence (Zielinski et al. 2019), also called the social costs (Brouwer et al. 2017). As the preference of beachgoers for a “clean beach” are usually the main reason for beach cleanings, environmental education and awareness-raising are central issues for the acceptance of beach management measures (Zielinski et al. 2019; Kataržytė et al. 2020). There is another aspect hindering their management procedure that beach wrack is often not yet included in international policies. Sometimes beach wrack is only covered as a side aspect by national or local regulations, e.g., for handling and recycling. Thus, problems of local municipalities range from losses of income to increasing costs and restrictions on handling collected material (Chubarenko et al. 2021). Despite its relevance in research and policies for decades, there is still a lack of a harmonized beach management and policy implementation within the Baltic Sea Region addressing sandy beaches adequately.

Consequently, beach management from a holistic perspective is needed which can be given by ecosystem service assessments that are explicitly anthropocentric. Ecosystem services (ES) are defined as the benefits humans obtain from ecosystems directly or indirectly (Costanza et al. 1997). The Common International Classification on Ecosystem Services (CICES V5.1) according to Haines-Young and Potschin (2018) and Maes et al. (2015) distinguishes the three main categories: provisioning, regulating and maintenance, and cultural ecosystem services. Due to the difficulty in assessing the value or the monetary background, “pure” ecosystem functions without direct or indirect benefits to humans are often neglected by the public. Studies include this aspect by adding a fourth category of supporting services (Millennium Ecosystem Assessment 2005) or the “ecosystem integrity” (Müller et al. 2020; Müller and Burkhard 2012). Widely accepted ecosystem service terminology differentiates between the potential (stock or potential supply), flow (actual use or real supply), and demand for ecosystem services (Burkhard et al. 2014; Müller et al. 2020). Scientific and political interest and relevance of ecosystem services increased exponentially during the last decades (Chaudhary et al. 2015; Bouwma et al. 2018). A vast range of assessment methods for modeling, mapping, and evaluation of ecosystem services exist based on biophysical, socio-cultural, and monetary values. A decision tree was developed by Harrison et al. (2018) to support the selection of appropriate methods depending on the purpose and available data input. Consequently, for management issues ecosystem service assessments can provide an integrated view that is needed to include stakeholders’ perspectives combined with biophysical data as well as economic consequences of measures.

Despite the often recreational focus at sandy beaches, they provide a wide range of ecosystem services. With regard to provisioning services, Emadodin et al. (2020) assessed the potential of beach wrack as agricultural fertilizer. Other studies on maintenance and regulating services range from coastal protection by wave attenuation (Defeo et al. 2009) to its potential as carbon sinks (Beaumont et al. 2014). Most studies focus on cultural services, for example evaluating the willingness to pay for beach ecosystem services (Enriquez-Acevedo et al. 2018) and the impact of beach wrack on tourism and bathing quality (Quilliam et al. 2015). Nevertheless, a holistic ecosystem service assessment of the overall provision of beach wrack and beach ecosystems is still lacking.

The prevailing question of this study is how marine litter and beach wrack affect Baltic sandy beach ecosystem services. As representative areas for high impacted beaches, we focused on sandy beaches in Germany and Lithuania. They exhibit different hydrodynamics (e.g., exposition, fetch), socioeconomic characteristics (e.g., population, uses, hinterland), and environmental conditions (e.g., seagrass meadows in front of the shore). The aims of this study were (1) to identify and assess the importance of ecosystem services for the overall provision at sandy Baltic beaches, (2) to develop beach scenarios that are representative of common Baltic beach management, (3) to assess the impact of beach wrack and litter on beach ecosystem services using two new remote and multidisciplinary expert-based assessment approaches, (4) to further differentiate between ecosystem service potential and flow by a combined data-based assessment approach, (5) to show trade-offs and synergies between beach management measures and give recommendations for improved beach management by showing its practical relevance, (6) to show applicability and opportunities of ES assessments within international coastal and marine policy implementation.

## Management of Sandy Baltic Beaches in Germany and Lithuania

**Fig. 1:**
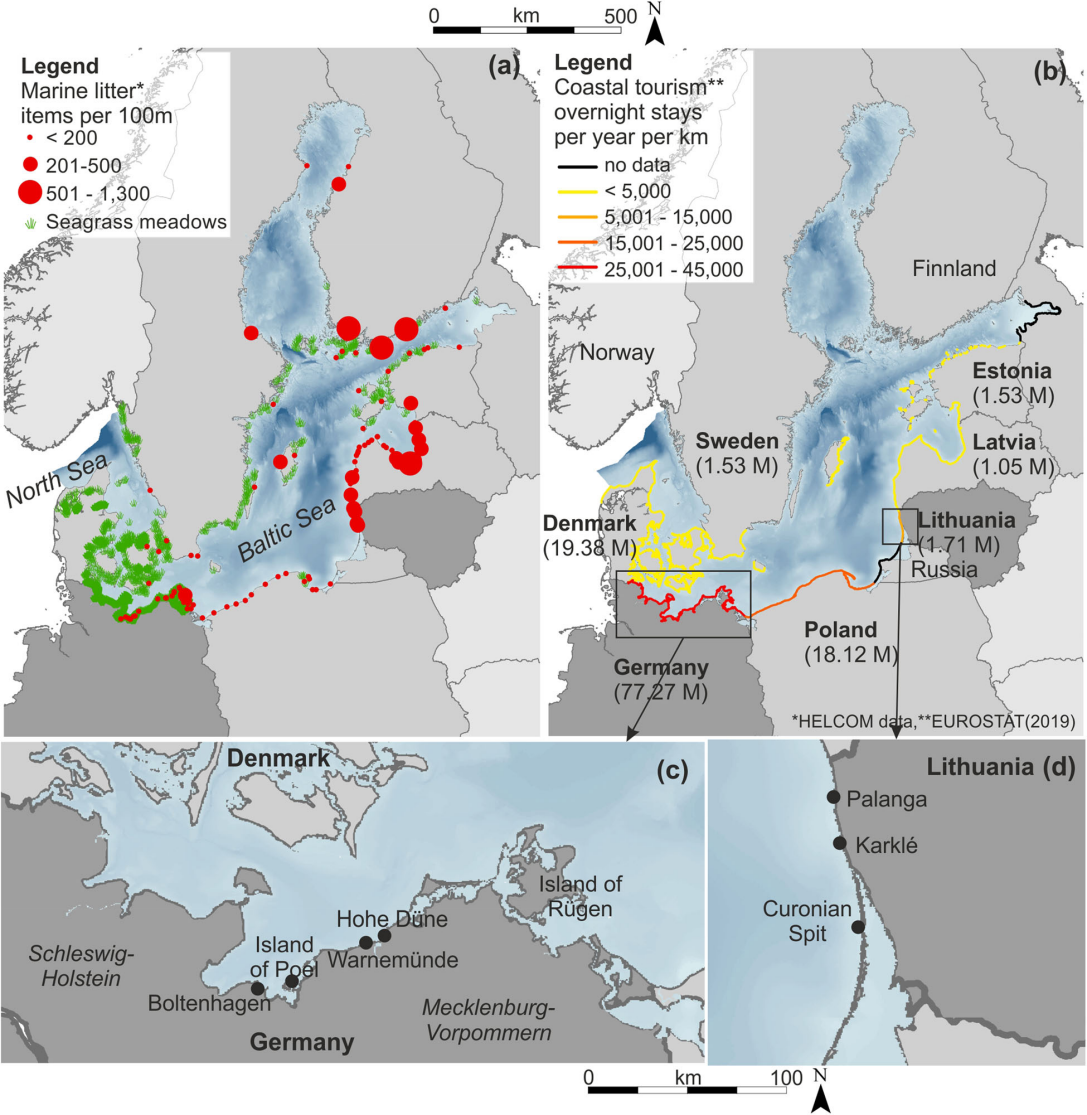
Map of the Baltic Sea indicating (**a**) marine litter distribution and seagrass meadows (HELCOM data), (**b**) coastal tourism in overnight stays in 2019 per km and in total (in brackets) (Eurostat 2019), (**c**) the German Baltic coast and (**d**) the Lithuanian coast with study sites

**Fig. 2:**
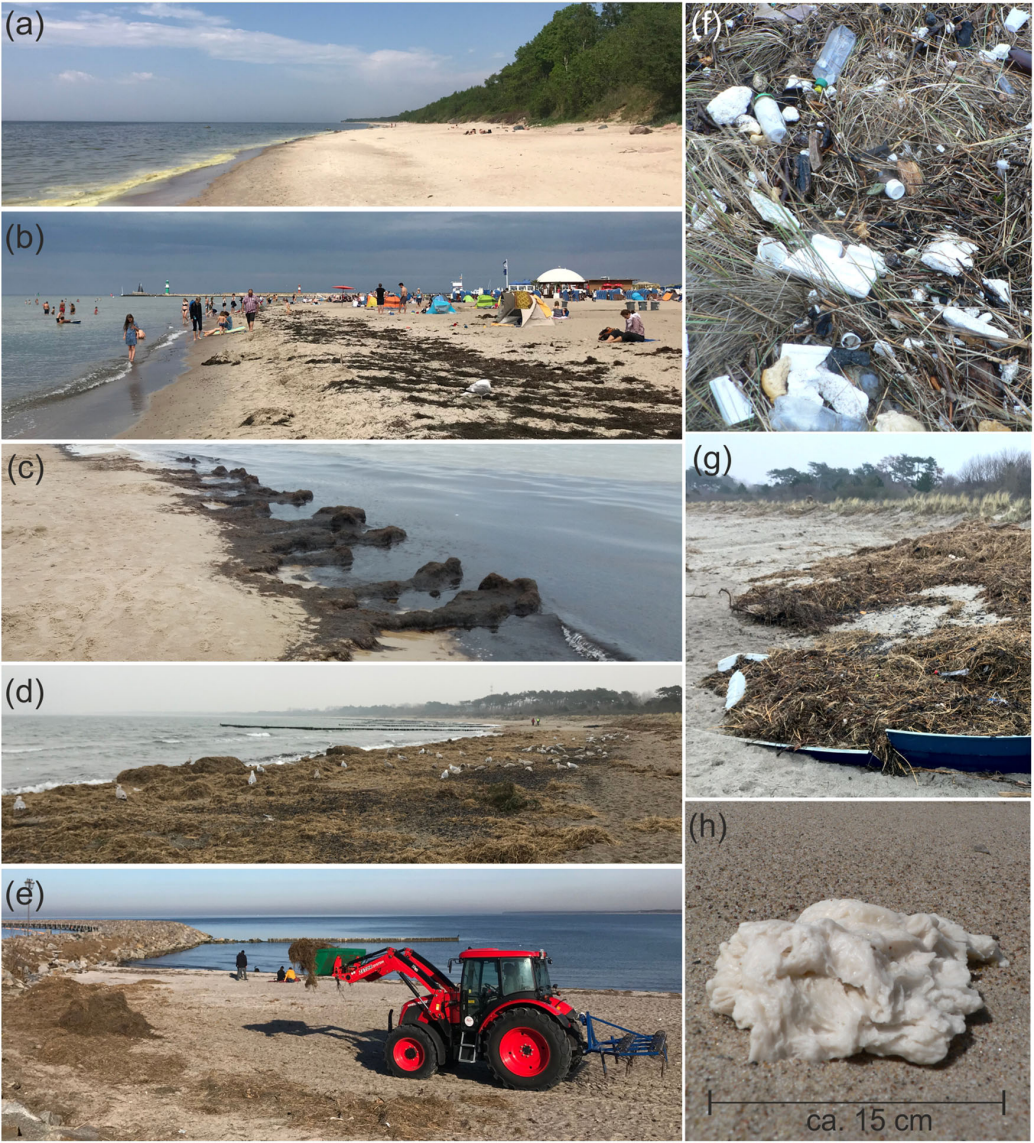
Sandy beaches in Lithuania (LT) and Germany (GER): (**a**) remote Karklė beach in the regional park (protected) in May 2019 (LT), (**b**) summer season in Warnemünde in 2020 (GER) not cleaned, (**c**) decomposing beach wrack in Palanga (LT) in October 2020, (**d**) beach wrack accumulation after a storm in January 2019 in Hohe Düne (GER), (**e**) a mechanical beach cleaning of beach wrack at Hohe Düne January 2019, (**f**, **g**) beach wrack mixed with marine litter, and (**h**) piece of paraffin wax at a beach of Curonian Spit (LT)

In the Baltic, Germany has by far the highest pressure from coastal tourism with 77.29 million overnight stays yearly (Eurostat 2019) (Fig. 1). The German Baltic outer coast has a length of 720 km including 450 km of sandy beaches (Kliewe and Sterr 1995), that are mainly of dissipative character due to hydrodynamic conditions as low water depth, low wave exposition, short fetches, and little slope (Froehle and Fittschen 1998). Only 22 km of the coastline is under nature protection including several Natura 2000 habitats and two national parks (Vorpommersche Boddenlandschaft and Jasmund) (Schumacher 2008). Due to dense populations of seaweed, beach wrack washed ashore mainly consists of eelgrass (mainly of *Zostera marina L*., rare *Zostera noltii Hornem*.) and brown algae (e.g., *Fucus vesiculosus L*.) (Chubarenko et al. 2021). Comparatively high amounts of up to 1,000 kg/m year^−1^ with an average of 269 kg/m of beach wrack (in total 4,900 t) are projected to accumulate annually (Mossbauer et al. 2012). Accumulation hot spots are more common at western beaches (e.g., Island of Poel or Boltenhagen), but also at piers or bights after storm events, for example at Hohe Düne with amounts of up to 20 kg/m per event (Fig. 2), or at the Island of Rügen with up to 1,000–2,000 t/ year^−1^ (Chubarenko et al. 2021). Beach litter pollution compose mainly of cigarette butts and plastics items (Haseler et al. 2017) showing a relatively low median value of 47 items per 100 m (OSPAR method) varying from 7 to 404 items (Schernewski et al. 2017) compared to Lithuanian beaches. During the summer season, beaches were cleaned mechanically and daily at beachside resorts (e.g., Warnemünde). Removed material amounted up to 269 kg/m on average beach wrack mixed with sand (Mossbauer et al. 2012). Beyond that, seasonal cleaning takes place when certain amounts of biomass accumulated, e.g., after winter storms (e.g., January 2019 Hohe Düne, Fig. 2). Costs sum up to annually 38€ per meter managed beach (Mossbauer et al. 2012), showing annual costs from 7.6–253€/m³, with the highest values in Scharbeutz of up to 140,000€ (Jensen 2017). Regarding legal aspects of handling and recycling opportunities, according to the German federal law (KrW-/AbfG section 3 part 1–circular economy/waste law), beach wrack that is accumulated on beaches is defined as organic waste, while also further direct use as fertilizer is strictly regulated.

With a length of 90.6 km, the Lithuanian coast is separated into the outer coast of Curonian Spit (50.0 km) mainly consisting of sandy beaches, and the mainland including beaches (38.4 km), moraine and sand cliffs (5.6 km), and natural coastal dunes (3.7 km) (Jarmalavičius et al. 2012). The coastline is highly exposed with long fetches. Coastal tourism in Lithuania counted up to 1.71 million overnight stays yearly (Eurostat 2019) (Fig. 1). As data on beach wrack composition is lacking, we assume the main composition of beach wrack is based on the described macroalgae communities such as *Polysiphonia spp*. (red algae), *Furcellaria lumbricalis* (red), *Cladophora spp*. (green) and a low amount of *Fucus vesiculosus* (brown) (according to studies of Labanauskas (1998), Bučas et al. (2007), and Bučas et al. (2009)). Comparatively low amounts of beach wrack at the beachside resort Palanga sum up to 400 t (Schultz-Zehden and Matczak 2012). Marine litter compositions show high amounts of paraffin wax (Fig. 2) also commonly accompanied by amber (Esiukova 2017; Haseler et al. 2017) with comparatively high mean values of 222 items per 100 m varying from 138 to 340 items (Schernewski et al. 2017). Beach wrack and marine litter management along the Lithuanian coast differ depending on the use and level of protection of the coast. In some sections, where it is a part of protected territory, especially in Seaside Regional Park, beach wrack and marine litter it is not removed and left to its natural conditions. At public beaches or recreational sections of the coast, beach wrack and marine litter is being managed based on municipality and public area cleaning company contracts, to ensure an attractive and clean environment for tourists. Around 45% of the coast is not managed due to the remoteness and no public use interest, mostly along the Curonian spit (~32 km). At the main beachside resort Palanga (Fig. 2), since 2019 daily mechanical beach cleaning takes place during tourism season from 15th of May to 15th of September (~40 moto-hours/month), while done before only manually or semi-manually. In 2019, a total of 1.49 t per 35 ha beach wrack and litter were collected. This resulted in an estimated cost of 32 €/m^2^ for beach wrack and litter removal.

## Methods

**Fig. 3:**
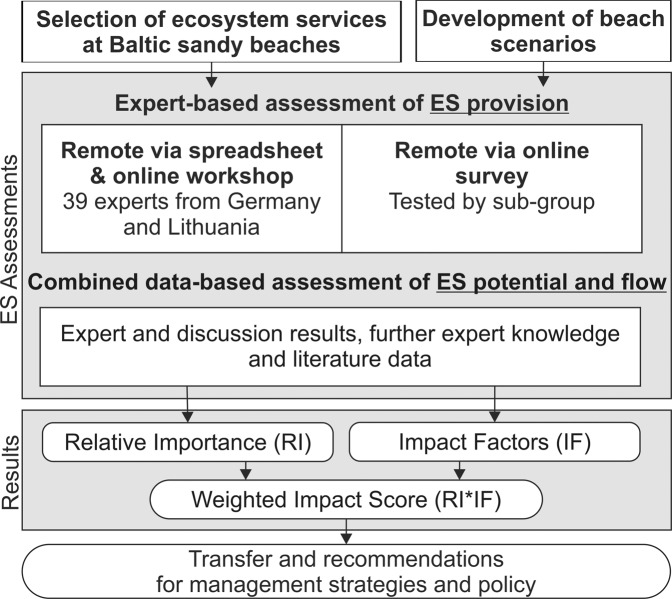
Flow diagram of study methods and assessment design

We first followed a two-steps preparation phase (Fig. 3). After selecting a set of ecosystem services explicitly for assessing southern Baltic sandy beaches, we developed four representative beach scenarios for the study area and their beach management. Based on these, two expert-based ecosystem service assessments were carried out to assess the relevance of beach ecosystem services as well as the impact of beach wrack and litter on such provision. Complemented by a combined data-based assessment we further differentiated between the general service provision, potential (stock or potential supply), and flow (actual use or real supply) to give recommendations for practical beach management and policy implementation.

### Selection of Ecosystem Services and Scenario Development

We selected a set of 21 services relevant for local management and policy specifically for Baltic sandy beach ecosystems (Table 1). These are based on the Common International Classification of Ecosystem Services (CICES V.5.1) according to Haines-Young and Potschin (2018), adapted from Müller et al. (2020) and Barbier et al. (2011). Description and examples are specified to the study area, southern Baltic sandy beach ecosystems, while services on the class level are generally valid for sandy beaches globally (Defeo et al. 2009).

**Fig. 4:**
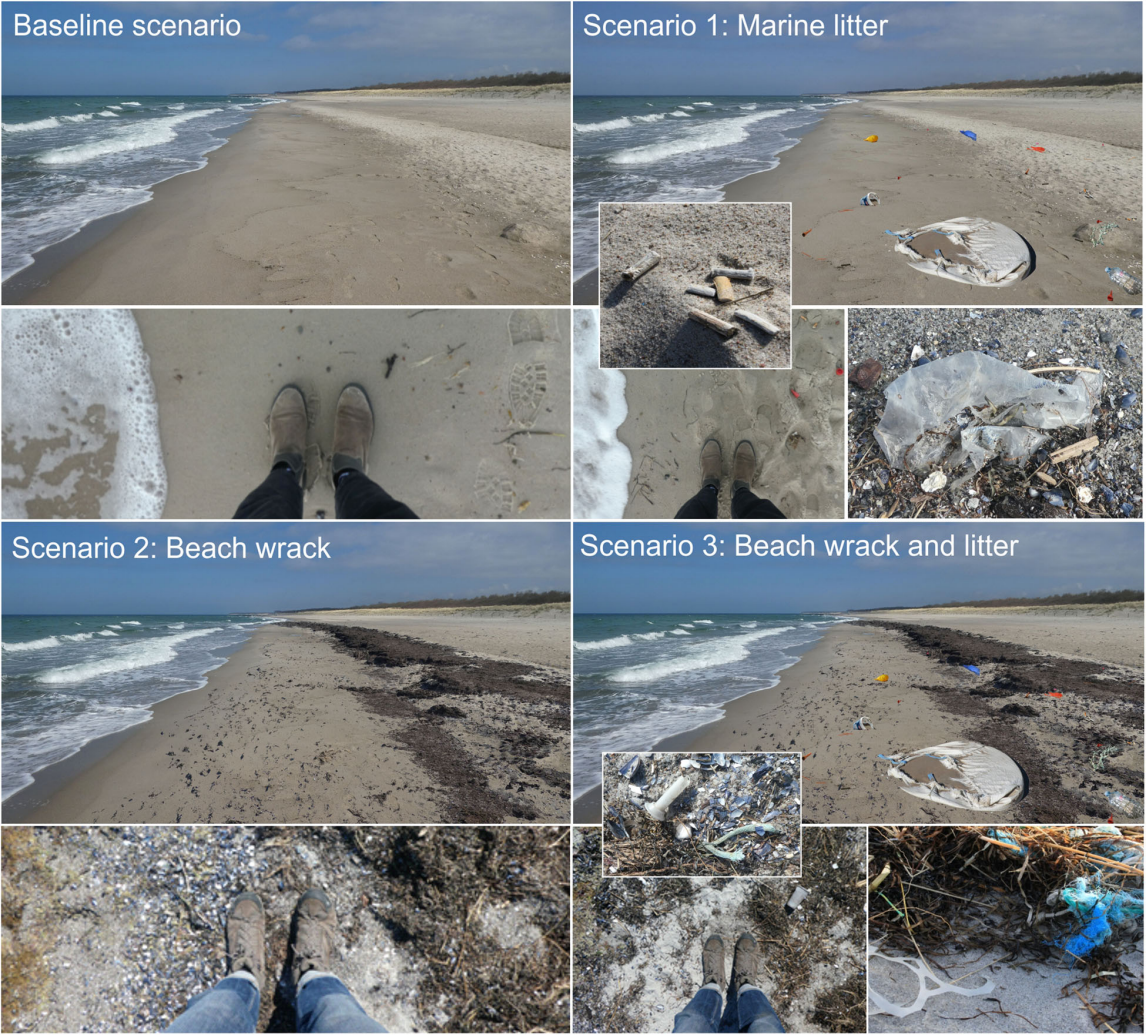
Visualization of four beach scenarios developed showing different states of beach wrack and litter accumulations

Four realistic beach scenarios were developed representative for common management measures in the Baltic as the basis for a comparative ecosystem services assessment (Fig. 4). The scenarios include different states of beach wrack and litter accumulations (excluding micro litter).

- *Baseline scenario*: shows a common Baltic sandy beach without accumulations of beach wrack nor marine litter. Thus, it is representative of beaches that look alike naturally with little to no wrack accumulation. Furthermore, it describes the state of art and most common management practice after cleanings (mechanically, manually by hand, or both) at beaches used for tourism.
- *Scenario 1*: shows marine litter accumulations from both the sea and land without beach wrack. It is defined by moderate to high amounts of marine and beach litter with around 300 macro litter items per 100 m beach length. It describes commonly polluted beaches in the vicinity of cities and human settlements.
- *Scenario 2*: shows beach wrack accumulations without marine litter. We defined a 35% coverage of beach wrack within 10 m from the swash zone to the beach (beach width). It describes near-natural beaches without cleaning measures, usually in remote areas without direct access or parking lots.
- *Scenario 3*: shows accumulations of both beach wrack (35% coverage within 10 m from the swash zone to the beach) and marine litter (~300 items). It describes beaches that are not regularly managed nor cleaned, for example, remote beaches, but also beaches after storm events.

### Ecosystem Service Assessments

We applied a multidisciplinary comparative ecosystem service assessment approach comprising three steps: (1) remote expert-based assessments via spreadsheets individually and online workshops in groups, (2) remote expert-based assessments via an online survey for a methodological test, and (3) a combined data-based assessment integrating expert values and discussion results, further expert knowledge and literature data (Fig. 3).

**Fig. 5:**
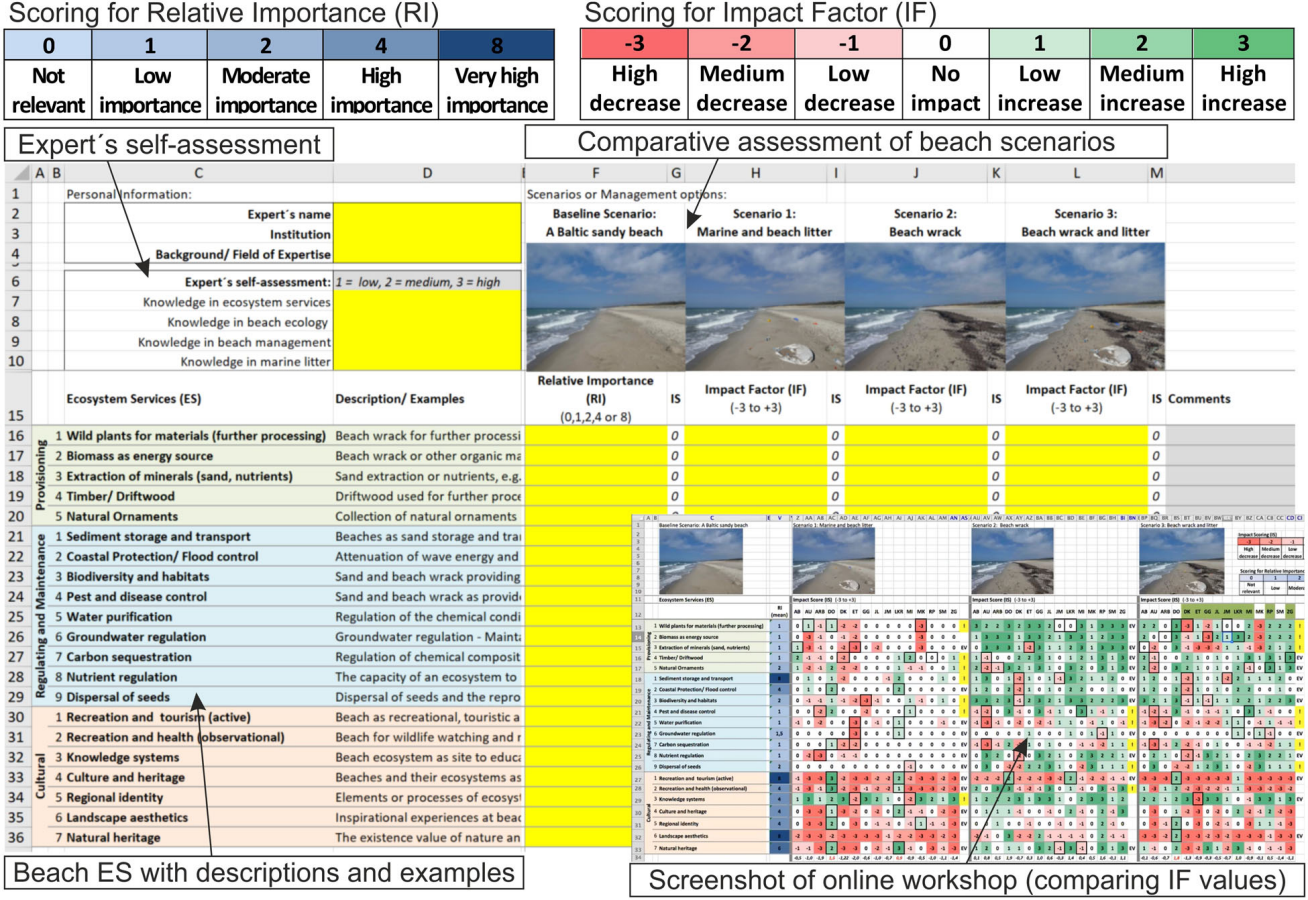
Design of expert-based assessment via spreadsheet including scoring for assessment and an exemplary screenshot of an online workshop

First, remote expert-based assessments via spreadsheets were carried out, based on an already tested comparative expert-based approach for coastal and marine ecosystem services (Inácio et al. 2018). We collected data through rating ecosystem services and assessing impacts by the developed beach scenarios. Assessment results showed perceptions, knowledge, and values of ecosystem services from different experts. A total of 39 experts replied to this spreadsheet-based assessment within a time span of 10 days, individually and remotely, supported by a guideline including detailed scenario description and edited photos accordingly (Fig. 5).

Experts assessed empirically the “Relative Importance” (RI) of each ecosystem service for the total provision at a Baltic sandy beach in general and independent from any scenario (Fig. 5). We used a non-linear scoring (0, 1, 2, 4, 8) to highlight extreme values in perceptions, for more robust and clear results, and to better differentiate between services. Furthermore, the suitability and handiness of the scaling and tool should support the experts during the assessment. Afterward, the experts rated the “Impact Factor” (IF) indicating the relative change or impact of each scenario compared to the baseline scenario. We used a scaling from high decrease (−3) to high increase (+3) in service provision based on experiences from former assessments (Schernewski et al. 2017).

During three online workshops on 4 June 2020, 19 June 2020, and 2 July 2020 experts discussed argued their given values and could modify them in case of misunderstandings (29 experts were present). Each workshop took around 90 min including an introduction, a presentation of preliminary results, and a structured discussion that was recorded (Fig. 5). The aims of the workshops were to discuss extreme values and outliers going through all services and scenarios addressing experts directly, to compile different argumentations and views, and to identify possible misunderstandings. Afterward, experts that could not attend were interviewed in additional and individual online meetings.

Experts were mainly scientists (31) from seven different universities and institutes, but also from non-governmental organizations and initiatives (7), other governmental institutions (1), and state authorities (1). Experts had different university degrees (bachelor, master, Ph.D., professor) from diverse disciplines (biology, ecology, geography, engineering, geoinformatics, numerical modeling, oceanography, coastal and marine management). In addition, we divided the experts into groups according to their institutional nationality: Lithuania (14) and Germany (25) and their level and field of expertise: ecosystem services (12), marine litter (13), ecology (14) based on their self-assessment and the authors’ estimate.

**Table 1:** Selected beach ecosystem services for the assessment of Baltic sandy beaches (derived from own data and based on CICES V. 5.1 according to Haines-Young and Potschin (2018), Müller et al. (2020), and Barbier et al. (2011))

		Ecosystem service classes	Description and examples
Provisioning (P)	1	Wild plants for materials (further processing)	Eelgrass for insulating material (e.g., in the construction and building sector)
			Eelgrass for stuffing material (e.g., pillows, mattress)
			Beach wrack as soil improver (e.g., in gardening and agriculture)
			Beach wrack as coastal protection (e.g., dune restoration)
	2	Biomass as an energy source	Beach wrack for energy conversion (e.g., bio gas or fuel, biochar)
	3	Extraction of minerals	Extraction of nutrients from beach wrack (e.g., as fertilizer)
			Sand extraction
	4	Timber/Driftwood	Driftwood used for further processing (e.g., handicrafts, arts)
	5	Natural ornaments	Collection of natural ornaments (e.g., seashells) washed ashore for arts, jewelry, and souvenirs
Regulating and Maintenance (RM)	1	Sediment storage and transport	Beaches as sand storage and transport for natural coastal dynamics
	2	Coastal Protection/Flood control	Attenuation of wave energy and flood prevention (e.g., beach width, inclination, vegetation, or beach wrack)
	3	Biodiversity and habitats	Beaches and their ecosystem providing suitable habitats and nursery grounds
	4	Pest and disease control	Beaches and their ecosystem as the provider of habitat for native pest and control agents (to keep the system´s resilience)
	5	Water purification	Regulation of the chemical condition of salt waters by living processes (e.g., algae, sea grass)
	6	Groundwater regulation	Maintaining of water cycle features (e.g., water storage and buffer, natural drainage, irrigation, and drought prevention)
	7	Carbon sequestration	Regulation of chemical composition of atmosphere and oceans by sequestration of carbon
	8	Nutrient regulation	The capacity of an ecosystem to store and recycle nutrients (e.g., nitrogen and phosphorus for beach soil and dune vegetation)
	9	Seed dispersal	Dispersal of seeds and the reproduction of lots of plants e.g., resuspension by beach wrack and natural coastal dynamics
Cultural (C)	1	Recreation & tourism (active)	Beach as recreational, touristic area (hiking, swimming sunbathing) and sports spots
	2	Recreation & mental health (observational)	Beach for wildlife watching and nature observation
	3	Knowledge systems	Education: Beach ecosystem as a site to educate about nature conservation and human-nature conflicts
			Research: topic and study object of interest
	4	Culture and heritage	Beaches and their ecosystems as part of cultural heritage, thus historically important (e.g., history of sailors and fishermen, seaside festivals)
	5	Regional identity	Elements or processes of ecosystems that contribute to a person’s individual identity (sense of belonging) or strengthen people’s group identity
	6	Landscape esthetic	Inspirational experiences at beaches and their ecosystems for enjoyment of nature (natural beauty)
	7	Natural heritage	The existence value or non-use of nature and species themselves, preservation for future generations

As a common method for uncertainty analysis, the Monte Carlo simulation test allowed us to compute randomly repeated samples of data to assess its patterns and diminish errors in sampled data (Carsey and Harden 2013). Computing random samples repeated certain times (10, 20, 30, 40, 100, 1000), we identified the number of experts to be at least 30 to achieve robust sampling data.

**Fig. 6:**
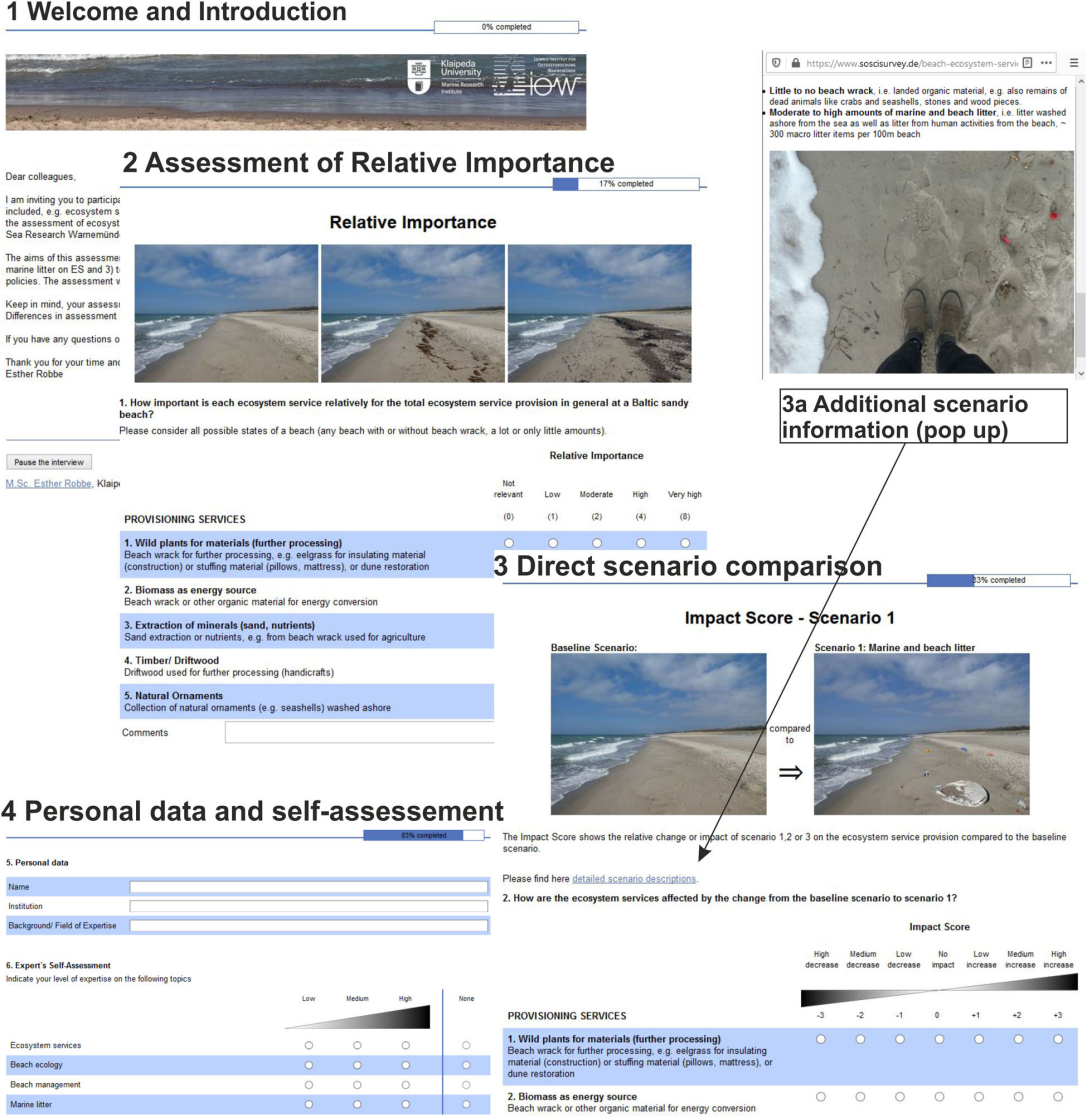
Design of expert-based assessment via online survey showing exemplary webpages

Secondly, for a methodological test and comparison, we applied the same approach via an online survey (www.soscisurvey.de) (Fig. 6). We tested and compared the applicability for the interviewer and the usability for the interviewees deducting strengths and weaknesses for both the spreadsheet-based assessment and online survey based on pre-defined indicators. These included technical setup and data analysis (interviewer) and comprehensibility, practicability, technical usability, and time requirements (interviewee). Five experts from the first group were asked to carry out the same assessment also via the online survey. Here we aimed to compare both methods and to give recommendations when and why to use which methodological implementation.

Thirdly, the main aims of the combined data-based assessment approach were to reduce subjectivity and bias of expert results, to fill knowledge gaps and clarify misunderstandings among experts, to confirm and compare experts’ and literature data (if existent). This assessment was carried out by the authors of this paper using expert values and discussion results, further expert knowledge, and literature data (Fig. 3). We also further differentiated between the potential supply or stock (here only referred to as “potential”) and real supply or actual use (here only referred to as “flow”) of beach ecosystem services for a more detailed view and possible use within coastal management. Furthermore, we combined all relative importance (RI) values with the impact factors (IF) calculating a weighted impact score (IS) by simple multiplication for comparison and the final assessment of both the expert-based and data-based results (Table 2).

**Table 2:** Results of expert-based (service provision) and combined data-based assessments (service potential and flow) showing Relative Importance (RI), Impact factors (IF), and the weighted Impact Scores (RI in % × IF = IS) for all three scenarios

Ecosystem services	Relative importance				1: Marine litter						2: Beach wrack						3: Beach wrack and litter					
					Impact factor			Impact score			Impact factor			Impact score			Impact factor			Impact score		
	Data	Experts	Data (%)	Exp. (%)	Potential	Flow	Provision	Potential	Flow	Provision	Potential	Flow	Provision	Potential	Flow	Provision	Potential	Flow	Provision	Potential	Flow	Provision
P1	1	1	1.3	1.5	0	0	0	0.0	0.0	0.0	3	2	3	4.0	2.7	4.4	3	1	1	4.0	1.3	1.5
P2	1	1	1.3	1.5	1	0	0	1.3	0.0	0.0	2	1	3	2.7	1.3	4.4	3	1	1	4.0	1.3	1.5
P3a	1	1	1.3	1.5	0	0	−1	0.0	0.0	−1.5	2	1	2	2.7	1.3	2.9	2	0.5	−1	2.7	0.7	−1.5
P3b	1		1.3		0	−1		0.0	−1.3	0.0	0	−1		0.0	−1.3	0.0	0	−1		0.0	−1.3	0.0
P4	1	1	1.3	1.5	0	−1	0	0.0	−1.3	0.0	0	−1	1	0.0	−1.3	1.5	0	−1	1	0.0	−1.3	1.5
P5	2	2	2.7	2.9	1	0.5	0	2.7	1.3	0.0	3	2	2	8.0	5.3	5.8	3	1.5	1	8.0	4.0	2.9
RM1	8	8	10.7	11.7	0.5	0.5	0	5.3	5.3	0.0	1	1	1	10.7	10.7	11.7	1.5	1.5	1	16.0	16.0	11.7
RM2	8	8	10.7	11.7	0	0	0	0.0	0.0	0.0	3	3	1	**32.0**	**32.0**	11.7	3	3	1	**32.0**	**32.0**	11.7
RM3	8	4	10.7	5.8	1	−1	−1	**10.7**	−10.7	−5.8	3	3	3	**32.0**	**32.0**	**17.5**	3	2	1	**32.0**	21.3	5.8
RM4	1	1	1.3	1.5	−1	1	0	−1.3	1.3	0.0	2	2	0.5	2.7	2.7	0.7	2	2	0	2.7	2.7	0.0
RM5	1	1	1.3	1.5	−1	1	0	−1.3	1.3	0.0	2	2	0	2.7	2.7	0.0	1	1	0	1.3	1.3	0.0
RM6	1	1.5	1.3	2.2	0	1	0	0.0	1.3	0.0	0	2	0	0.0	2.7	0.0	0	2	0	0.0	2.7	0.0
RM7	1	1	1.3	1.5	0	0	0	0.0	0.0	0.0	−1	−1	0	−1.3	−1.3	0.0	0	0	0	0.0	0.0	0.0
RM8	4	1	5.3	1.5	−2	−2	0	−**10.7**	−10.7	0.0	3	3	2	16.0	16.0	2.9	3	1	1	16.0	5.3	1.5
RM9	1	2	1.3	2.9	1	−1	0	1.3	−1.3	0.0	2	2	2	2.7	2.7	5.8	2	1	1	2.7	1.3	2.9
C1	8	8	10.7	11.7	0	−2	−2	0.0	−**21.3**	−**23.4**	0	−2	−2	0.0	−**21.3**	−**23.4**	0	−3	−3	0.0	−**32.0**	−**35.0**
C2	8	4	10.7	5.8	0	−1	−2	0.0	−10.7	−11.7	3	2	2	**32.0**	**21.3**	11.7	3	1	−1	**32.0**	10.7	−5.8
C3	2	4	2.7	5.8	1	1	1	2.7	2.7	5.8	1	1	2	2.7	2.7	11.7	1	1	2	2.7	2.7	11.7
C4	4	4	5.3	5.8	0	−2	−1	0.0	−10.7	−5.8	0	2	0	0.0	10.7	0.0	0	−1	−1	0.0	−5.3	−5.8
C5	2	4	2.7	5.8	0	−2	−1	0.0	−5.3	−5.8	0	1	0	0.0	2.7	0.0	0	−1	−1	0.0	−2.7	−5.8
C6	8	8	10.7	11.7	0	−2	−3	0.0	−**21.3**	−**35.0**	0	−1	0	0.0	−10.7	0.0	0	−3	−3	0.0	−**32.0**	−**35.0**
C7	4	4	5.3	5.8	0	−2	−2	0.0	−10.7	−11.7	0	2	1	0.0	10.7	5.8	0	1	−1	0.0	5.3	−5.8
Sum					2	−12	−12	11	−92	−95	29	26	24	149	124	75	31	11	0	156	34	−42

Bold values show the highest impact scores for each scenario, indicating also the main trade offs between services (positive and negative values in bold)

## Results

### Relative Importance (RI) of Beach Ecosystems Services—Expert-Based

**Fig. 7:**
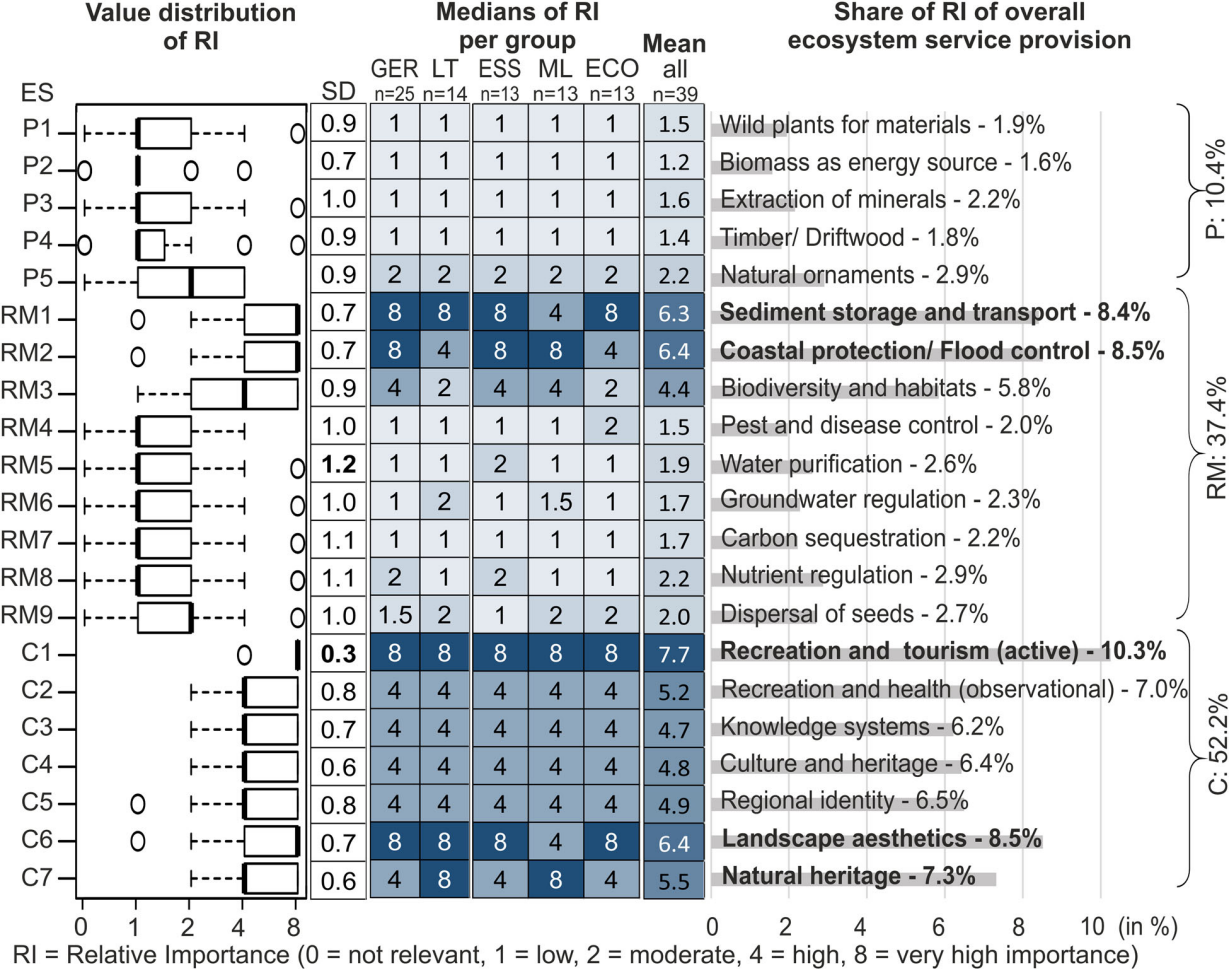
Expert-based results on the Relative Importance (RI) for provisioning (P), regulating and maintenance (RM) and cultural (C) services [standard deviations (SD); institutional nationality (GER Germany, LT Lithuania); field of expertise (ESS ecosystem services, ML marine litter, ECO ecology)]

The most relevant category with 52.2% were the cultural services, showing high (4) to very high importance (8) for all services (Fig. 7). Three services of the regulating and maintenance category (37.4%) are of high importance (8) (RM1, RM2, RM3), while all provisioning services (10.4%) indicated only low (1) to moderate (2) importance.

The highest agreement among experts accompanied by the lowest standard deviation (SD) was calculated for cultural services (0.64) while provisioning (0.88) and regulating and maintenance services (0.97) represented a higher dispersion. Excluding two NVs (no value), the relatively spontaneous assignment of values was sustainably changed by subsequent discussions in the workshops: 14 from 39 experts changed 67 out of 817 values (8.2%) (in detail see Supplementary Information).

Only 6 out of 21 services (29 %) differed across institutional nationality. More differences were seen across fields of expertise, 10 out of 21 services (48%), mainly among “Ecology” and “Marine litter” groups (9 services). However, the largest difference is only one class of change (e.g., low to moderate). With regard to the respective expertise level (bachelor, master, Ph.D., prof) only the value estimation for one service, biodiversity, and habitats (RM3), differed significantly. Thus, the variability of assessment values among expert groups based on institutional nationality, field, and level of expertise was low.

Perceptions on the importance of provisioning services (=P) were partly based on different interpretations of ES terminology (potential vs. flow) as well as of definitions and descriptions (Fig. 7). High values for wild plants were stated due to the interpreted potential for the further economical processing of the material (P1). Instead, historic flows were the primary reason behind low values. Similarly, biomass as an energy source (P2) was assessed of high importance due to its potential, but limited by high energy loss and economic costs in material managing. Low values for mineral extraction (P3) from the collected material likely stated a lack of such practices at our study sites. Mineral collection in Germany and Lithuania takes place either off-shore or from inland deposits. A historic potential of amber included as a mineral by some experts may be a reason behind higher values. Driftwood (P4) was sometimes understood as marine litter, while others did not consider it as a beach wrack component nor marine litter (despite guideline definition). Experts mainly mentioned amber and seashells for collecting natural ornaments (P5). Nevertheless, experts emphasized that in Germany and Lithuania it is legally forbidden to take natural resources from the beach if it is not for personal use.

Background for different perceptions on the importance of regulating and maintenance services (=RM) was also partly interpretations of ES terminology (supply and demand). The services sediment storage and transport (RM1) and coastal protection (RM2) became more relevant with increasing demand, which highly depends on beach exposure and location. The low value for a variety of species at sandy beaches resulted from the experts’ lack of knowledge and comparison to other habitats (e.g., forests or meadows). Compared to the otherwise relatively species-poor sand areas of beach ecosystems, beach wrack and the drift line were seen as biodiversity hotspots, representing pristine and unique habitat characteristics (RM3). Results indicated low importance for pest and disease control (RM4). However, experts’ interpretations ranged from threats to human health to ecosystem level, e.g., including the fact that sometimes beach wrack is enriched with aggregated filaments of drifting harmful microalgal blooms. The capacity of sand as a filter for water purification (RM5) was considered mostly irrelevant, as there is only retention of coarse and solid material, and sand only contains small amounts of organic matter. Due to the high porosity of sand grains and consequently a lacking water storage capacity, some experts pointed out a possible enhancement of saltwater intrusion of groundwater (RM6). Low values for carbon sequestration (RM7) were estimated due to the low binding of carbon to a corresponding matrix, e.g., plants. An exception is the dunes, which have a higher potential to store carbon by their vegetation. The carbon content in beach wrack was indicated to be relevant only with regard to further storage or processing by management activities. Experts evaluated nutrient regulation (RM8) to be of low to moderate importance when removed beach wrack biomass was assumed to be further processed on land, e.g., as compost. Furthermore, the same importance was given for the beach wrack biomass when left at the beach (within or across habitat level). The dispersal of seeds (RM9) was assessed as not relevant seawards and at more exposed beaches, but of low importance when considering their dispersal onshore via sand movement (from shore to dunes).

Recreation and tourism (C1) and recreation and health (C2), mentioned here as cultural services (=C), are very common (e.g., sunbathing, sports), popular, and an important economic factor in the Baltic region. Furthermore, beaches are also used for education and science (C3) with their diverse ecosystem characteristics and issues. A similar important is culture and heritage (C4), which includes for example public sea-side festivals and sailors’ tales. Regional identity (C5) is explained as the feeling of belonging or being at home in a particular region or desire to live next to the sea and coast. Landscape esthetics (C6) as a personal perception of beauty is regarded as a prerequisite for most of the cultural services. As a natural heritage (C7), people want to preserve beach ecosystems for future generations.

### Impact Factor (IF) of Beach Scenarios on Service Provision—Expert-Based

**Fig. 8:**
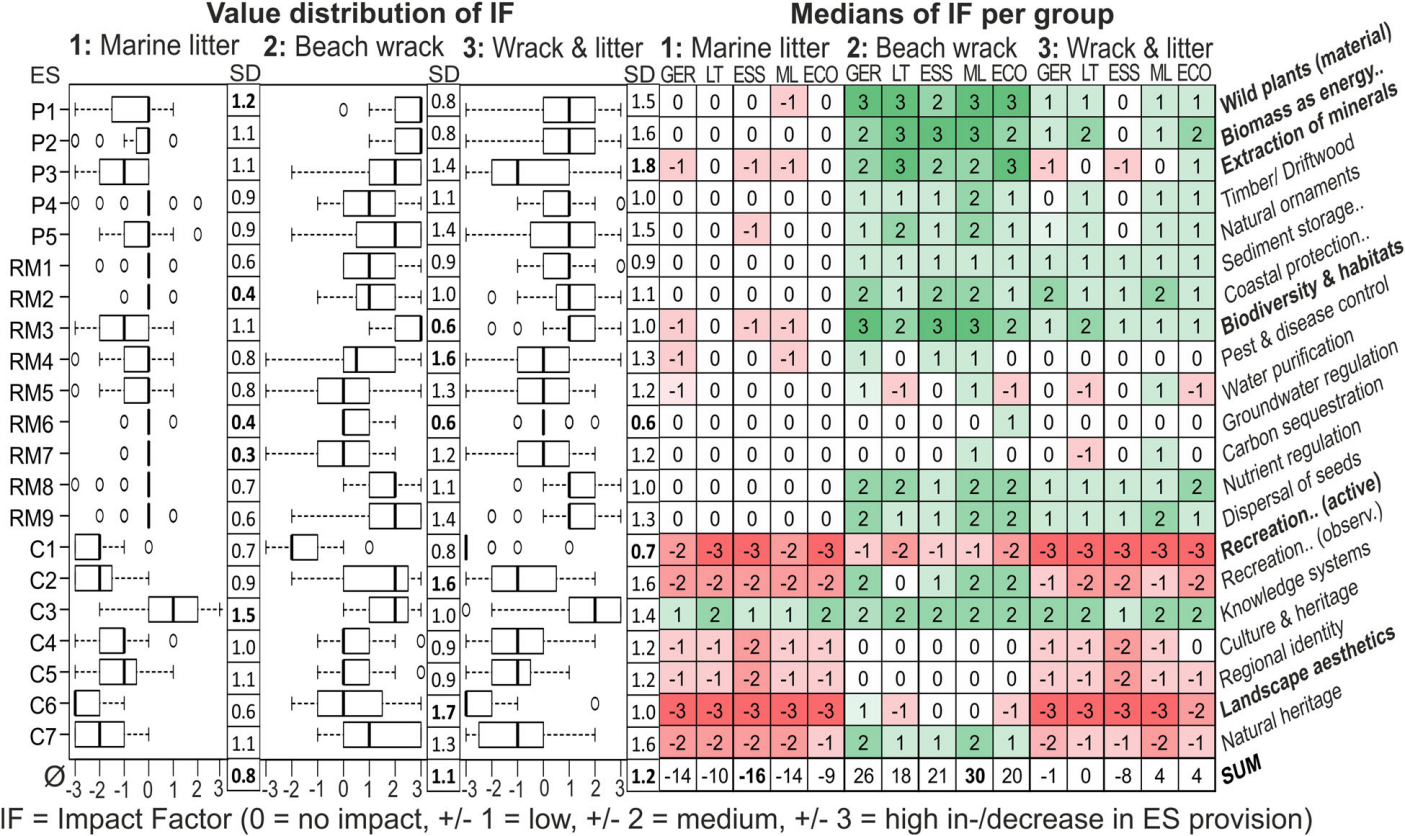
Expert-based results on Impact Factors (IF) of scenarios for provisioning (P), regulating and maintenance (RM) and cultural (C) services [standard deviations (SD); institutional nationality (GER Germany, LT Lithuania); field of expertise (ESS ecosystem services, ML marine litter, ECO ecology)]

Litter affected all cultural services negatively but one (C3), while the remaining services only showed low to no impact in service provision by litter only (scenario 1) (Fig. 8). Contrarily, beach wrack affected all provisioning, regulating, and maintenance services positively apart from one service (RM5), while results for cultural services are inconsistent including both positive and negative impacts. Among all services, only one cultural service (C1) was affected negatively. Other cultural services indicated no to moderate positive impacts except from one (C6) showing inconsistent values. Litter added to beach wrack (scenario 3) had the most negative impact when compared to scenario 2 and scenario 1 for all provisioning, regulating, and maintenance services (mainly P1-3, RM3). However, within mixed compositions, the negative impact of litter prevails for most cultural services, while the positive impact of beach wrack prevailed for most provisioning, regulating, and maintenance services.

Experts revealed the highest agreement, or lowest standard deviation (SD = 0.8), for the addition of litter as a clear negative impact trend on service provision. Most disagreement among experts is shown for both beach wrack scenarios 2 (SD = 1.1) and 3 (SD = 1.2). From 2457 IFs excluding six NVs (no value), 16 experts decided to change 186 values after discussion (7.6%) (in detail see Supplementary Information). This indicates that results are robust and valid also without discussions. Only two services exhibited inconsistent results including positive and negative IFs (RM5, C6). As only a few services indicated two classes of change, mainly on expertise level, differences among expert groups are very low.

Main reasons for the dispersion of RI and IF values are based on different interpretations regarding (1) ecosystem service terminology (i.e., potential or actual use, supply, and demand), (2) definition and description of services (sand and nutrients as minerals), (3) reference frame (within or across habitats, long or short-term perspective, size classes of litter, sea or land), and (4) due to misunderstandings and lack of knowledge (scenario descriptions, driftwood as marine litter, pests as risk for human health), and additionally (5) subjectivity (mainly cultural services), and (6) field of expertise and institutional nationality.

#### Scenario 1—Marine litter accumulated at the beach

Different perceptions about litter accumulations at the beach are partly based on misunderstandings, assuming for example beach wrack presence or dune vegetation used as biomass for further economical processing (P1) (Fig. 8). Experts argued that litter could serve as additional energy input within incineration plants (P2). They expected that the use and processing of sand (P3) and of collected driftwood (P4) were more challenging when contaminated with litter, e.g., due to necessary separation before use. Some experts assumed similar drifting characteristics of litter and driftwood, thus a correlation of landed material, which explains the positive outlier and values. Tourists would be discouraged by a high percentage of litter in their collection of natural material and would prefer cleaning activities (P5). Others assessed the potential that would not change or even increase when considering litter also as natural ornaments (e.g., art projects or collecting sea glass).

Experts identified litter (especially bigger items) to serve as an erosion catalyst, or as additional physical barriers to trap sand within a small scale and short-term perspective (RM1-2). Others argued that there was no impact at all, as the amount of sand remained the same and litter presence is too low. Regardless of its texture, experts stated that litter as a hard substrate added to the ecosystem can serve as additional habitat for organisms, e.g., crabs using litter as a refuge or epiphytes for fixation (RM3). They also assumed that litter poses a danger to wildlife by simple entanglement or as nesting material of birds. More severe pollution was expected by an increased accumulation and breakdown of litter in smaller fragments over time. Litter was also seen as a carrier or habitat for pathogens, pests, or invasive species (RM4). Consequently, experts expected an increased demand for pest and disease control which will be correlated with litter amounts. Possible harmful pollutants out of litter could be released within ambient water. However, experts assumed a higher impact on the ecosystem, when the material is defragmented into micro up to nano-size level because this increases the uptake by organisms as well as the surface area for colonization (RM5-6). Only a few experts considered litter as possible carbon sequestration or as a release of carbon via decomposition processes (RM7). Most experts expected that litter does not affect the recycling of nutrients, unless if higher concentrations of pollutants are introduced into the system (RM8). For the dispersal of seeds and similar to RM4, some experts regarded litter as an additional hard substrate. It could serve as a carrier for seeds and seedlings as also for bacteria and viruses considering different terrestrial and aquatic-influenced transmission paths in water and air (RM9). Litter may also hamper seeds in germination and growth, from dispersal (e.g., trapped in a bottle) or avoid growth by covering areas (i.e., obstacles to wind or wave-driven dispersal).

Perceptions of litter impact on cultural services differed mostly due to subjectivity and the experts’ perspective—their own or as common tourist. Litter is a clear nuisance to beach tourism (C1-2), that is impacted as soon as the esthetic sentience is affected. They assumed litter to have a negative impact on beach goers’ sensitivity to uncleanliness in particular (C2). Litter was expected to increase the visibility of human-nature conflicts which is being used for educational and scientific purposes and awareness-raising (C3). As an additional parameter, as a manmade problem, litter within the ecosystem does not reflect pristine natural conditions, thus changes research discussion and experimental designs by altering the study of natural ecosystem processes and functions. Litter might cause a decrease in the sense of personal identity by shame and embarrassment (C4-5). However, litter can present historical conditions for later archeological research about our current lifestyle or serve as inspiration for art projects. Experts also argued that pollution could lead to a strengthening of group identity via activism and personal engagement (e.g., “clean up” activities). Litter is a strong visual nuisance for enjoyment and perception of a pristine nature (C6). Higher litter amounts increase the desire to keep the environment intact and conserve it for future generations, even though some argued that marine litter does not impact the actual value of nature significantly (C7).

#### Scenario 2—Beach wrack accumulated at the beach

Perceptions on organic biomass amounts at the beach (beach wrack) differed mostly based on interpretations of ES terminology (Fig. 8). For example, the actual use (or flow) of wild plants for further processing, e.g., of respective species like eelgrass or brown algae (P1) or as biomass for energy production (P2) was assessed as very limited or unknown. However, since there is currently an increase in public environmental awareness, the economic potential as a resource for e.g., building insulation or as an initial biomass supplement for biochar/biogas was expected to increase in the future. For sand extraction (P3) the moisture level and the amount/composition of biomass were considered as challenges for further use, as the meshes of sieves of the machines were clogged with the sand-biomass mixture. Regarding the use of beach wrack for soil improvement and fertilizing, e.g., for gardening/agricultural purposes, a higher proportion of organic biomass is a prerequisite. Amber also catches far better in stranded seaweed thus more amber can be found here. Consequently, due to a better trap function and similar buoyancy (thus drifting characteristics), most experts expected a positive correlation between amounts of beach wrack and driftwood (P4) as well as natural ornaments (P5). However, beach wrack might also cover or entangle driftwood and natural ornaments, causing higher efforts to collect.

Most experts emphasized that higher beach wrack accumulations reduce erosion even in front of the beach, as they attenuate wave energy and contribute to sand trapping. The accumulation zone at the beach can serve as a further sand trap for wind-driven particles both from sea and land and might broaden the beach area (RM1-2). However, in relation to the larger scaled sediment transport along the coastline and its physical processes, beach wrack was mentioned as a minor impact. Experts assessed beach wrack as an important habitat and consequently hot spot for biodiversity, e.g., microbiological processes (e.g., bacteria) and organisms like invertebrates, insects, and birds (RM3) (cf. “Selection of Ecosystem Services and Scenario Development”). Some indicated the possible occurrence of potentially toxic microorganisms and pathogens within beach wrack, while others mentioned the disease-reducing function as a habitat for native pest control agents (RM4). When pest probability is increasing, some experts assumed that biotic interaction or feedback and thus the capacity of pest control also increases accompanied by higher demand for this service. Due to leaching, a release of nutrients and potential pollutants out of beach wrack were expected to enter the water (RM5), which could enhance eutrophication. Experts suspected that wet beach wrack close to the water line is releasing higher concentrations of nutrients and possibly harmful substances than dried beach wrack at the upper beach area close to the dunes. The decomposition of organic material emits greenhouse gases like carbon dioxide and methane (RM7). Consequently, experts indicated that the removal and further processing of beach wrack could reduce these emissions and thus improve carbon storage capacities. Beach wrack as a major nutrient source for the ecosystem was identified as the basis of life at beaches (RM8). Organic matter is an important nutrient source (i.e., phosphorus and nitrogen) for the early stages of soil formation in dunes, but might enrich parts of the coastal forest or salt meadows as well (cf. “Selection of Ecosystem Services and Scenario Development”). Experts also discussed the possibility of beach wrack removal as an easy and cheap way for remediation of the Baltic Sea. Similar to litter, beach wrack was mentioned as a trap, as seeds and seedlings could be entangled and/or transported over long distances (RM9). However, the organic matter could function as an accumulation matrix, it protects seeds from being simply drifted. Dispersal is probably more successful in larger accumulations while protecting seeds from washing away accompanied by an enhanced attraction for animals feeding and further dispersing (e.g., birds).

Similar to litter, perceptions of beach wrack impact on cultural services differed, within the expert group, mostly due to subjective opinions or perspectives. Experts estimated beach wrack concordant as disturbing and a nuisance to beach tourism (C1). This perception likely depends on the location, characteristics, and infrastructure of the respective beaches. Beach wrack could be a disservice to human recreation due to its strong smell during decomposition. Some people are also scared of algae aggregated in the water or at the beach, as they assume it causes allergies, is unhealthy, or the touch creates a bad feeling. Contrarily, experts mentioned that beach wrack accumulations support a higher animal density and thus a better possibility to observe nature and wildlife (C2). Since only scarce ecological studies about its spatial and seasonal composition and respective amounts of beach wrack along the Baltic Sea exist, experts considered beach wrack as an interesting topic for further research and also for education (C3). Only a few experts assessed an impact on landscape esthetics (C6), but as beach wrack is mostly removed from touristic beaches, it was assumed that the “common” beach tourists notice high amounts of beach wrack negatively. Beach wrack accumulations were estimated to increase the intrinsic existence value of beach ecosystems, thus the value of nature (C7).

#### Scenario 3—combined beach wrack and litter accumulation

Further economical processing of beach wrack is hampered when mixed with litter (especially plastics) (Fig. 8), as experts expected collection and separation need more effort and hence is not cost-efficient (P1-2) (cf. “Ecosystem Service Assessments”). Consequently, contamination of beach wrack with litter decreases biomass quality and usability. Similarly, both sand extraction and further use as fertilizer or soil conditioner are hindered by litter (P3).

Some experts considered litter only as a minor impact on biodiversity and habitat conditions when mixed with beach wrack (RM3). Furthermore, they stated that invertebrates, insects, and birds still inhabit polluted beach wrack even though life within the habitats is affected by pollutants and danger of injury. For pest and disease control (RM4) the negative impacts of litter on survival were balanced by the positive impact of beach wrack as an additional food source and habitat. As also mentioned for P1-3, the amount of litter reduced the potential use of beach wrack and hence the impact on nutrient regulation (RM8) as well as for dispersal of seeds (RM9).

Similarly, impacts on recreation and tourism (C1) were expected even stronger when beach wrack and litter is mixed (−3). For recreation and health (C2) experts argued that positive and negative impacts were off-setting, as beach wrack increased wildlife biodiversity and litter disrupted the “natural appearance”. However, the negative litter influence appeared much stronger. As the only exemption among cultural services, namely knowledge systems (C3), impact factors changed only slightly. For culture and heritage (C4) and regional identity (C5), impact factors of beach wrack mixed with litter were almost identical to those of litter only (scenario 1). Impact values of −3 for landscape esthetics (C6) indicated that litter with or without beach wrack represented always a strong negative impression. In relation to the respective single components, for natural heritage (C7) the negative impact of litter prevailed within the mixed composition.

### Combined Data-Based Assessment and Weighted Impact Score (IS)

The combined data-based assessment differentiated further between the potential and flow of beach ecosystem services (Table 2). This differentiation is necessary due to hidden data when only assessing the general provision, e.g., by off-setting effects due to different interpretations of terminology or services (i.e., contradicting values). Especially when using the results as an indicator for decision-making, further differentiation is necessary complemented by the weighted impact score for direct comparisons. Furthermore, hereby we aimed to reduce subjectivity and bias of expert results, to fill knowledge gaps and clarify misunderstandings among experts, to confirm and compare experts´ and literature data (if existent). Additionally, based on discussion results and off-setting effects for extraction of minerals (P3) we divided this service further into sand and nutrient extraction.

Comparing RI values, there were only minor differences between expert and data-based results. Anyway, the importance of 3 out of 7 cultural services shifted slightly, while 4 out of 9 regulating and maintenance services gained 20% in importance with the combined approach. The main difference can be seen for nutrient regulation (RM8) which shifted from low (1) to high (4) importance in the combined assessment.

The litter had only little impact on the service potential (no change for 59% of services), but impacted highly the flow (sum moduli: 21), mostly negative (sum: −11) and mainly on the cultural services category. Consequently, experts who assessed the impact of litter (with and without beach wrack) on the general service provision (sum: −12) were mainly referring to the flow. Beach wrack instead exhibited a strong positive impact for both potential and flow (sum: 31 and 28). Compared to the service provision, experts were referring mostly to the potential of beach wrack. All cultural services were affected on the flow level, while only 24% of the values on their potential were impacted by beach wrack and/or litter.

We now calculated the weighted impact score (IS) by multiplying the relative importance (RI) in % with the impact factors for each service and scenario. Thereby, impacts are only considered according to their relevance. Consequently, impacts on services with high importance are considered stronger. With the impact score, we created an indicator for decision-making within coastal management which can be used to compare service categories, individual services, and scenarios. Furthermore, trade-offs and synergies among services can be identified by their negative and/or positive IS. Scenarios 2 and 3 (beach wrack with and without litter) showed main trade-offs between two regulating and maintenance services (RM2-3) and two cultural services (C1, C6) at flow and provision level (positive and negative IS > 30). There are only small trade-offs on potential level (±10.5), here mainly for two regulating and maintenance services (RM3, RM8) impacted by litter (scenario 1).

Several studies state the high potential (+3) (Table 2) of further economical beach wrack processing (P1), e.g., as insulation material for construction, filling material for pillows, and use for dune restoration (Sterr et al. 2019; Chubarenko et al. 2021; Misson 2020). However, due to still an unprofitable processing and additional litter pollution, the flow was assessed only low (+1) to moderate (+2). Studies show a moderate potential (+2) for energy conversion of beach wrack, e.g., as a substrate for biogas plants (Barbot et al. 2015) or biochar production (Misson 2020), while the flow increased only a little (+1) due to its low competitiveness, for example, with energy crops. Current studies emphasize the innovative potential (+2) of removed beach wrack for further use, thus extraction of nutrients (P3a). For example, they propose further use as nutrient-rich fertilizer processed in reed bed systems (Kupczyk et al. 2019) or as high-quality organic fertilizer (Emadodin et al. 2020). Seasonal variability of beach wrack biomass in composition and amounts, and increased costs for further use as fertilizer due to additional effort of litter separation were indicated by a low increase in service flow (+1). In Germany and Lithuania, sand extraction for several construction measures (P3b) is done commonly by seafloor dredging and terrestrial sand mining, but no sand extraction is performed at the coast or beach (Staudt et al. 2019; Pupienis et al. 2014). Litter nor beach wrack impact the service potential (0), but if the sand is contaminated, it needs to be cleaned before further use or processing (flow: −1). As recent literature neglects a correlation of beach wrack (incl. driftwood) and litter at Portuguese sandy beaches (Guerrero-Meseguer et al. 2020), the potential of driftwood (P4) was assessed as not impacted (0). Nevertheless, the flow decreased slightly (−1), as biomass collection and separation need more effort. The service for so-called natural ornaments (P5) included litter items as well, for example, commonly collected sea glass and other items used for art or awareness-raising projects, resulting in a positive impact on service potential (+1) and flow (+0.5). However, higher biomass landings of beach wrack also indicate a higher content of natural ornaments (potential: +3) for the interested target group (Esiukova 2017), while also being a possible nuisance due to smell or entanglement (flow: +2).

Landing of sand and beach wrack in areas with lower currents enables the spreading of larger beach areas like a “storage” (RM1), enriching fore-dunes via eolian transportation and providing additional material dissipating wave energy (potential and flow: +1) (Everard et al. 2010). Concerning coastal protection (RM2), Nordstrom et al. (2011) presented in their study the importance of beach wrack for eolian sand transport, as it is acting like a sand trap, thus influencing the sediment budget and formation of the fore-dune and its crest (potential and flow: +3). Indicated by the negative impact on service flow (−1), litter can be ingested by marine organisms and birds, used as nesting material, and cause entanglement of wildlife (Kühn et al. 2015) (RM3). A low increase in potential (+1) revealed its function as an additional hard structure for new habitats of marine organisms (Kiessling et al. 2015). Other studies indicate that beach wrack (potential and flow: +3) support a rich supralittoral fauna (Defeo et al. 2009) and emphasize their high importance as habitat and food source for dominant species at sandy beaches, e.g., sandhoppers (Ruiz-Delgado et al. 2016; Pelletier et al. 2011). Pest and disease control (RM4) was moderately impacted by beach wrack on their service potential and flow (both +2). However, beach wrack might include harmful substances, but it is a matter of concentration. One important ecological function of organic matter is to maintain the balance and capacity to control pests and diseases due to several decay processes. Plastics were identified as possible carriers of pathogens, harmful microalgae, and invasive species (Audrézet et al. 2020; Kiessling et al. 2015). Keswani et al. (2016) mentioned litter as a possible biotope for spreading further fecal indicators (FIOs) and harmful algal bloom species (HABs). Therefore, even though the potential (−1) slightly decreased, due to a higher demand for this service the flow (+1) increased slightly. With respect to a study of Everard et al. (2010), where sand dunes were mentioned as actively managed parts of the water purification (RM5) infrastructure in Amsterdam for supplying drinking water, we assumed that this service (“Selection of Ecosystem Services and Scenario Development” and “Ecosystem Service Assessments”) is only relevant when considering sand dune systems. However, studies on groundwater regulation (RM6) at sandy beaches and within dunes are lacking. Defeo et al. (2009) stated that water storage in dune aquifers and groundwater discharge through beaches is one relevant ecosystem service at sandy shores. With the low importance (1) of this service due to research gaps, the potential could be underestimated. The process of carbon sequestration (RM7) at sandy beaches compared to other ecosystem services and habitats like forests or wetscapes are only of low importance (1) for ecosystem service interpretation. If sand dunes are included in the analysis, the potential would increase, as the plants of vegetated dunes and adjacent coastal forests are able to sequester carbon at a rapid rate (Beaumont et al. 2014). However, several studies reported that beach wrack might be a significant source of greenhouse gas emissions (GHG) like carbon dioxide and methane (Misson 2020; Rodil et al. 2019; Goméz et al. 2018). In conclusion, beaches as land-sea interface possibly play a more important role in carbon cycling than expected. Dugan et al. (2011) showed in their study that sandy beaches play an important role (RI:4) for nutrient regulation (RM8). As primary producers like micro- and macroalgae or seagrass grow in nearshore waters and use nutrients, their service potential is correlated with processing and re-mineralization of organic material and accumulation of dissolved nutrients. Other studies also emphasize the importance of sandy beaches for nutrient cycling across habitats (Barreiro et al. 2013; Rodil et al. 2019; Gómez et al. 2018). Litter was assessed to increase the service potential slightly (+1) of dispersal of seeds (RM9), while the flow was decreasing due to possible entanglement (−1) (Kiessling et al. 2015).

Litter and beach wrack presence and amounts are a common reason for the visitors’ choice of their beaches (Zielinski et al. 2019; Kataržytė et al. 2020). Consequently, for recreation and tourism (C1) moderate (−2) to high (−3) impacts were stated, while the service potential is not impacted (0). With increasing infrastructure and consequently paid spa taxes, acceptance of both beach wrack and litter decreased at German Baltic beaches (Borcherding 2020). They also found a positive correlation between the awareness of the ecological relevance of beach wrack and its public acceptance, which justified the high potential (+3) but only moderate flow (+2) of the service recreation and health (C2). Humans perceive beaches and coasts as very valuable for knowledge systems (C3) that consist of educational and awareness-raising activities (RI:2). The service potential and flow increased (+1) due to activities like beach clean-ups or nature observation hikes, but also scientific studies on beach ecology and the impact of litter (Hartley et al. 2018). Coasts are also highly important (RI = 4) in terms of being part of culture and heritage (C4) as well as (regional) identity (C5). Due to subjective perceptions, they are only impacted on the flow level (litter: −2 and beach wrack: +2/+1). Litter appeared to moderately (−2) disturb the service flow of landscape esthetics (C6), which is of very high importance (RI:8) for coastal regions (Corraini et al. 2018; Hartley et al. 2018). Studies also indicate that coasts are important as a legacy to preserve for future generations (RI:4), thus protecting natural heritage (C7) (Hartley et al. 2018).

### Methodological Comparison—Spreadsheet vs. Online Survey

For a methodological test and comparison of the spreadsheet-based method and the online survey, we aimed to assess both methods by pre-defined criteria in order to give recommendations when and why to use which methodological implementation (Table 3).

**Table 3:** Methodological comparison of expert-based ecosystem service assessments via spreadsheet and online survey

	Spreadsheet	Online survey
Technical set up *(Interviewer)*	Less time effort required At least basic software skills (e.g., Excel) required	Basic programming skills recommended (html, php)
Data analysis *(Interviewer)*	Easy data compilation for groups up to 50 experts (otherwise macros possible requiring programming skills) Easy and fast visualization of results for expert discussion	More complex data compilation (extraction from webpage and translation necessary)
Comprehensibility *(Interviewee)*	Additional guideline necessary (pdf)	Step-by-step guidance through webpage
Practicability *(Interviewee)*	More analytical details and information available (formulas, direct calculations of weighting factors, accumulated impact score) Easy and fast comparison of scores between scenarios (horizontal comparison)	Separate and direct assessment of scenarios (no misunderstandings or wrong comparisons) Common type of questionnaire (already used to) More difficult to compare and change score between scenarios No direct visualization of results or own interpretation possible
Technical usability *(Interviewee)*	Internet-only for down-/upload needed IT device needed (only computer) Spreadsheet software needed (excel recommended, but also usable with open source) Basic spreadsheet skills needed	Internet access needed IT device needed (computer, tablet or smartphone) No additional software or skills needed
Time requirements *(Interviewee)*	30–60 min (highly depends on commenting behavior)	15–45 min (highly depends on commenting behavior)

The assessment design of the spreadsheet and online survey was not identical due to the technical setup and differences in the software used (Table 3). The main differences in the online survey were step-by-step guidance through the whole assessment (page-by-page). Scenarios were compared individually and directly with the baseline scenario one after another. The main strength of the spreadsheet-based assessment is its fast and easy technical setup, while the online survey requires more time for implementation. On the other hand, while the assessment via the online survey can be done easier and faster, the spreadsheet-based assessment requires some more time from the experts.

## Discussion

### Ecosystem Service Assessment Approach—Methodology and Application

Within the expert-based assessment, specific ecosystem service terminology such as potential, flow, and demand (Burkhard et al. 2014; Müller et al. 2020) (cf. “Introduction”) was intentionally avoided by leaving the respective interpretation to the experts. Thereby, we gathered different arguments, understandings, and perceptions. Afterward, with the combined data-based assessment for a more ecological perspective, we further differentiated into service potential and flow. This approach allows for direct comparison of certain management scenarios, e.g., cleaning methods.

Comparing results of individual experts, values differed strongly, partly along with the whole range of values (from −3 to +3) (Fig. 8). Consequently, individual results were not representative or reliable especially with regard to beach wrack scenarios. In contrast, litter as a man-made problem was assessed very homogenously. However, results compared among expert groups, based on institutional nationality, educational background, and level, revealed low variability (low standard deviation/SD). Thus, our results indicated that a small number of experts within one group (*n* = 13) already showed representative results for the overall assessment (*n* = 39) (Fig. 8). Similarly, Campagne et al. (2017) calculated a minimal number of 30 experts needed for panel discussions in their ecosystem services study. This was also confirmed by our Monte Carlo simulations run beforehand that demanded at least 30 experts for our assessment design (cf. “Method”). However, expert groups should represent diverse institutions, levels, and fields of expertise equally, preferably a minimum of 10 experts per group.

Despite the differences between individual experts, our experience was that neither the institutional nationality nor the educational background and level within our expert groups significantly influenced the results. Although the assessment was specifically tailored to the Baltic Sea, it is, therefore, possible to transfer it to other beach ecosystems and local case studies, i.e., in the Mediterranean, provided that the scenarios used are realistic for these regions.

Furthermore, results showed possible bias due to different interpretations and misunderstandings, i.e., of definitions (e.g., beach wrack) and the descriptions of services (Table 1). Some experts, for example, also included driftwood in the beach wrack biomass or considered amber a mineral and not a natural ornament. Others interpreted pest and disease control with regard to human health and not as defined only for the ecosystem functioning itself leading to stronger perceived impacts. Some services indicated a need for further differentiation of their specific uses, as impacts were off-setting or contradicting. For example, while litter can decrease the potential of polluted beach wrack when used as a soil improver or fertilizer, for insulating materials there is no change. Besides, experts took different reference frames into consideration causing inconsistent data. For example, variability in results based on assessing the impact on services within and across habitats (only beach or including dunes and hinterland, sea and/ or land), on long or on short-term perspective, and on different size classes of litter (macro, micro, nano-level). Off-setting effects and biases within the assessment design may be caused by the selection and wording of services, their descriptions as well as the definition of the study area.

Jacobs et al. (2015) address trade-offs and synergies between ecosystem services using a matrix approach. The main synergy was found between biodiversity and recreation, while the main trade-off was identified between biodiversity and water use for navigation. Another study also assessed the relevance of single ecosystem services for different management scenarios (Schernewski et al. 2017). However, in our study we went one step further, assessing the relative importance of each service for the overall provision at beaches and using this for calculating a weighted impact score (IS). This allows us to compare the change among and between scenarios, as well as to show trade-offs and synergies among them. It can be easily adapted to local beach management by defining their local relative importance. Thus, the impact score (IS) is a suitable indicator for decision-making within practical beach management implementation by directly comparing different measures and identifying trade-offs and synergies in the Baltic and similar beach ecosystems.

Technically, the spreadsheet tool is most suitable for expert-based assessments, while the online survey is more suitable when addressing different stakeholders and larger groups of participants, e.g., “the general public”. Additionally, the combined assessment is needed for further, detailed ecological analysis and as a possible indicator for decision-makers. For participatory stakeholder engagement and consensus building, we recommend the general notion of “provision”, which necessitates a group discussion. In contrast, working with experts, we suggest using the terms “potential” and “flow” or “provision” when further differentiating in a combined data-based assessment.

### Beach Ecosystem Services—Relevance and Impacts

Results of this study showed that cultural services are the most important ones for the overall provision of ecosystem services at sandy Baltic beaches (52.2%) (Fig. 7). This can be partly explained by the assessment design, which is an entirely anthropocentric conceptual framework and thus targeting specifically human benefits derived from the ecosystem functioning. Furthermore, the photo-based visualizations helped to reduce bias by ensuring similar interpretations by the experts. This was important because amounts of beach wrack and its composition can vary strongly among seasons, years, and countries depending on currents, wind, and vegetation. Also, the location of beach wrack at the beach itself can highly influence the results, e.g., smelly and decomposing material near the coastline versus already dried out and partially buried in the sand in front of the dunes. Therefore, a joint understanding based on manipulated photos was crucial. However, the visualizations could also lead to an intrinsic bias towards cultural and provisioning services as they mostly represent visible elements of the ecosystem. Consequently, the expert-based assessments were likely too narrow and too biased for decision-making as a stand-alone, which emphasizes the relevance of further integration of biophysical parameters. However, it can serve as a basis for further in-depth analysis on the most relevant and/or impacted services. Especially for beach management purposes and for tackling man-made problems, our approach is a suitable attempt to weight and present the visible as well as the invisible values of sandy beaches and their ecosystem services.

Despite the low to moderate importance (37.4%) (Fig. 7) of regulating and maintenance services, the cultural services highly depend on and interact with them as underlying or supporting services (Kandziora et al. 2013). For example, bathing tourism requires functioning services like Baltic Sea remediation and water purification (i.e., bathing water quality). Nature observation walks also demand an intact ecosystem with wildlife and biodiversity. Thus, although only a few regulating and maintenance services were rated as highly important (RI:4 to 8) (Table 2), they play an essential role in securing ecosystem functions and thus for overall service provision.

There was a consistent agreement among experts on the high importance (RI:4 to 8) of cultural services. Instead, the impact factors varied much more, indicating disagreement about the extent and impact of litter and beach wrack on such. Especially when assessing cultural services (specifically C2 recreation and health, and C6 landscape esthetics), the experts’ subjective perspective affected the results. Impact factors differed considerably (covering 86% of the total range) when comparing respective opinions, for example, of a nature-lover, bird photographer, or hiker to a common beachgoer interested only in recreation and bathing. Some tourists prefer bare sandy and clean beaches, while others appreciate natural beaches with beach wrack. If not sought in a stakeholder workshop, this type of subjectivity could probably be reduced through an indicator-based assessment using socio-economic and biophysical data (Inácio et al. 2018; von Thenen et al. 2020). Another reason for high-value distribution is the low consent within the group, which can also show possible knowledge gaps or lack of understanding. Subjectivity among cultural services and general value distribution of RI and IF results indicated a need for and can be used as a spectrum for awareness-raising activities, adjusted provision of information, and moderation among different stakeholders’ perspectives.

Common beach management activities at Baltic sandy beaches reviewed in Borcherding (2020), Zielinksi et al. (2019), and Mossbauer et al. (2012) include different cleaning procedures. They differ with regard to the spatial area (flood accumulation zone, patches) and beach size, amounts, and composition of beach wrack and littering. Other important parameters are weather conditions (dry or wet sand), financial budget, and technical equipment and staff (heavy machinery, manually by hand, semi-manually). Based on these, the municipality thus determines the temporal frequency of the cleaning (daily, weekly or less, seasonal). Major criticism by nature conservationists (besides the removal of beach wrack and litter) is the use of heavy machinery that has an impact on the sediment characteristics and vegetation. This lead to compaction of the sediments/soils and the destruction of the fragile seedlings by the sheer weight of the machinery exerting enormous pressure on upper beach layers (Gheskiere et al. 2005). While there are no studies that focus specifically on the mechanical impact of beach cleaning vehicles, evidence for the disturbance of beach ecosystems through recreational driving with off-road vehicles on beaches is well established (Houser et al. 2013). Sand-dwelling microorganisms and invertebrates were hampered e.g., in the construction of new living tubes, and/or existing ones were destroyed. They are therefore no longer able to live in the swash area as a habitat or, if possible, have to retreat to not disturbed sections of the beach. This in turn affects the abundance and biodiversity of the species that feed on the inhabitants of the beach wrack infauna by depriving them of their food source (Defeo et al. 2009). However, intensive human use of beaches usually has already a strong impact on beach ecosystems, e.g., disturbances due to high trampling intensity by beachgoers (Seer et al. 2015). Hence, in high season it seems not to make a difference in the cleaning technique if cleaned manually or mechanically, while the distance to the next parking, and thus good accessibility, has an even higher impact (Borcherding 2020). Thus, our results can be interpreted as the impact of litter and/or beach wrack removal from the beach regardless of the cleaning technique and their impact and only considering a hypothetical removal. Consequently, our results are representative and can be used for applied beach management in the study area.

However, we also determined trade-offs between the removal of beach wrack and litter and the provision of ecosystem services (Table 2). For example, cleaning procedures usually also remove sand that can be hardly separated on-site when mixed with wet beach wrack. Consequently, the services sand storage (RM1) and coastal protection (RM2) are reduced due to the loss of sand. Another main trade-off refers to biodiversity and habitat, as by removing beach wrack also valuable habitats as well as the function of seed dispersal (RM9) are lost. Central trade-offs of beach cleanings (removal of wrack and/or litter) were identified for regulating and maintenance services, mainly coastal protection and biodiversity, and cultural services, mainly tourism and recreation, at flow and provision level. This indicated that beach management, or beach cleaning, mainly impacts the flow level, but not the potential.

Furthermore, we assume some synergies of tourism-driven beach cleanings, thus the removal of beach wrack and litter, that mainly intends to increase the cultural services (mainly C1, C6) (Table 2). Our data show possible synergies with provisioning services, as the collected material might be used further (P1, P2, P3). Nevertheless, when combined, the technological and economic feasibility of such seems to be very limited and of low potential. Furthermore, we estimate another synergy of beach wrack and litter removal for carbon sequestration (RM7) that might be increased or decreased by management techniques, e.g., storing beach wrack in dune systems or further use and processing, thus avoiding decomposition on-site causing greenhouse gas emissions. Furthermore, by removing beach wrack and litter, nutrients and/or heavy metals/pollutants that would harm the environment can be removed easily (RM5, RM8). In conclusion, beach cleaning can achieve several synergies through the removal of beach wrack and litter for further processing or for the purpose of providing services (e.g., soil fertilization, energy production).

### Transmission to and Recommendations for a Sustainable Beach Management

#### Remove litter, leave wrack

Based on our results and shown trade-offs, the removal of beach wrack is not favorable with regard to the overall service potential and flow, while the removal of litter can lead to an increase in the overall flow (Table 2). For beach management, it is therefore generally recommended to leave beach wrack on sandy beaches where it has landed naturally (if not posing an environmental or health risk), while it is strongly recommended to remove litter with as little shear pressure as possible, e.g., by manual collection.

#### Minimize the impact of cleaning

Despite our findings in favor of not removing beach wrack, site-specifics of beaches remain a major issue. For example, societal competitive pressures prevail on high tourism beaches. This leads to the conclusion for beach managers to carry out beach cleanings specifically on highly preferred and already degraded beaches due to strong human pressures (e.g., trampling intensity, pollution). To lower the impact of beach cleanings, new innovative techniques are needed. So far, light machinery or manual cleaning in reduced spatio-temporal patterns (e.g., only on-demand, in patches) are recommended.

#### Use as a valuable resource

The removed organic material is a valuable natural resource. Thus, we recommend using the synergies shown in this study and to support a value-adding process and use of the material. Depending on the composition, quantity, and quality of beach wrack, there are different forms of applications ranging from formerly known and reinvented to new and innovative ways of utilizing. These include beach wrack as filling material for pillows, as a soil improver and fertilizer, but also among others the use of biomass for energy conversion, for coastal protection and dune restoration, or as an insulating material for buildings.

#### Internalize (indirect) costs of cleaning

However, we also considered the high direct costs for beach cleanings (e.g., staff, machinery, maintenance) as well as the indirect “costs” by decreasing overall ecosystem service provision. Despite a possible loss of income from tourism caused by “polluted” beaches, the removal of beach wrack mainly affects the coastal protection function, the uptake and regrowth of dunes, and beach stabilization. In the long run, beach wrack removal is therefore not favorable in economic terms, as costs for future generations to protect and conserve their coasts and beach ecosystems are increasing. Thus, we recommend internalizing these indirect costs of beach cleanings, for example via taxes and fees following the ‘polluters pay principle’.

#### Increase awareness and environmental education

According to our results, the potential of cultural services at sandy beaches is less impacted by beach wrack and litter than the flow or provision (Table 2). This discrepancy between the combined data-based (potential) and expert-based results (provision) indicated a lack of awareness of the ecological value of beach wrack among our experts. Thus, we recommend implementing management strategies that are targeting awareness-raising and environmental education of beach wrack and its ecology, especially with regard to its function within sand dune formation and coastal protection. Thereby, the acceptance and understanding of beach management measures (less or no cleaning) can be increased through higher acceptance of beach wrack.

## Conclusion

This paper has argued that the removal of beach wrack at Baltic sandy beaches is not favorable with regard to the overall ecosystem service provision, as it has a strong positive impact on both service potential and flow. Contrarily, the removal of litter can increase the service flow significantly. In any case, synergies can be found in the cleaning of beaches heavily used for tourism by removing beach wrack for further processing or use (e.g., soil fertilization, energy production). Nevertheless, there are trade-offs between recreation and tourism, i.e., tourism-related removal of beach wrack, and the overall provision of ecosystem services at the beach, mainly coastal protection and biodiversity. The study contributes to our understanding of the interaction of management and policy measures with beach ecosystems and their services. Target audiences can vary from the general public to stakeholders and experts, depending on the purpose, which ranges from participatory stakeholder engagement to consensus building and decision making. This study is the first holistic assessment of ecosystem services provided by sandy beaches in combination with beach wrack and marine litter.

The findings and methodological approach will be of main interest to beach managers and policymakers in the Baltic Sea, but may also be applied and transferred to other beaches in the world showing similar characteristics, e.g., the Mediterranean Sea or the Black Sea. However, the visualizations used make the findings less generalizable, but the study can be repeated easily using photos and experts from new target regions. A limitation of this study is the geographical scope, as it did not cover services provided by dunes nor the near-shore water area explicitly, which could be usefully explored in future research. A further study could assess the impacts of concrete management measures and techniques applied by local municipalities, e.g., different machineries, by hand, or in patches. A challenge now is to develop new and innovative beach cleaning techniques and procedures, as well as economically feasible processing and application of beach wrack, which accumulates at beaches in different amounts, compositions, and high seasonality. Greater efforts of local authorities are needed to develop clear policy and legislation for sustainable beach and beach wrack management. Moreover, more guidance and consultation from research should be integrated into the decision-making of beach managers and policymakers. The approach used may also be applied to management issues in the context of coastal engineering and protection measures (e.g., hard and soft measures, building with nature), biodiversity and habitat management (e.g., recovery of seagrass meadows) or to support specific policy implementations (e.g., acceptance or monitoring of measures, define reference conditions or target values).

## Supplementary Information

Supplementary information
